# Urinary microbiome and urological cancers: a mini review

**DOI:** 10.3389/fruro.2024.1367720

**Published:** 2024-03-08

**Authors:** Gianmarco Randazzo, Eleonora Bovolenta, Tommaso Ceccato, Giuseppe Reitano, Giovanni Betto, Giacomo Novara, Massimo Iafrate, Alessandro Morlacco, Fabrizio Dal Moro, Fabio Zattoni

**Affiliations:** Department of Surgery, Oncology and Gastroenterology, Urologic Unit, University of Padova, Padua, Italy

**Keywords:** urinary microbiome, bladder cancer, prostate cancer, renal cell carcinoma, UTUC (renal pelvis and ureter), microbiome and dysbiosis, BCG (Bacille Calmette-Guérin)

## Abstract

**Introduction:**

The urinary microbiome (UMB) includes living bacteria, their genomes, and their products from interactions with the host environment. A “core” UMB could potentially exist, with variations between age and sex groups. Changes in UMB composition have been associated with benign urological disorders, but also with urologic cancers. Mechanisms through which UMB can trigger and maintain cancer can be local inflammation and interaction with immune system.

**Aim of the study:**

To describe the association between UMB and development of urologic cancers.

**Methods:**

A non-systematic literature review identified recently published studies (last 5 years), involving human patients, dealing with UMB. The database used for this review was PubMed, and the identified studies served as the base for a narrative analysis of the literature that explored the potential associations between UMB and urological cancers.

**Results:**

In bladder cancer (BC), UMB may play a role in epithelial-mesenchymal transition (and thus to progression to metastasis), as well as in effectiveness of BCG response rate. BC is also associated with changes in UMB, with bacterial richness indices increased in cancer groups compared to non-neoplastic groups and being different between NMIBC vs MIBC patients. In prostate cancer (PCa), there is an abundance in proinflammatory bacteria and uropathogens. In regard to renal cell carcinoma (RCC), penile cancer and testicular cancer there are still too few studies to draw significant conclusions about its relationship with the UMB.

**Conclusions:**

Gaining a deeper understanding of UMB role in urologic tumors could aid in the development of new therapies and improve classification of patients’ risk.

## 1 Introduction

The urinary microbiome (UMB) is a comprehensive concept that includes living bacteria, genes, genomes (identified through 16S ribosomal RNA sequencing), and their products resulting from interactions with the host environment (1, 2). UMB variability is assessed using alpha diversity (diversity of microbial populations within a single sample) and beta diversity (across multiple samples) (2–4). Lewis et al. (5) suggest the existence of a “core” UMB but other studies note variations based on age and greater heterogeneity in bacterial genera among females, with Actinobacteria and Bacteroidetes being more prevalent (5–7). UMB in females shares species and features with the vaginal microbiome, forming a connected system distinct from the gut microbiome (8).

Dysbiosis of the UMB has been associated to various urological disorders, including benign conditions such as interstitial cystitis (9), urgency urinary incontinence (10) and overactive bladder (11), but it also has been associated to prostate cancer, especially in a recurrent antibiotic exposure-setting (12). Moreover, a case-control study showed that regular probiotic intake reduced the risk of bladder cancer in the healthy population (13, 14), suggesting a possible association between UB and bladder cancer. In fact, in the last few years, several studies of UMB have shown potential associations between dysbiosis of UMB and the development and persistence of urological cancers, similarly to what happens for gut microbiome (15–17).

The UMB can influence the host tissues in different ways. Bacteria that are present in the urine can reduce ingested nitrates into nitrites; the formation of endogenous N-nitrosamines in the bladder leads to the initiation of neoplastic events in the cells. The carcinogenic effects of these compounds are related to the ability of the reactive chemical species alkylating microscopic constituents of organs (18–20). Epithelial–mesenchymal transition (EMT) is a series of molecular mechanisms that promotes metastasis in several cancers by detachment from basement membrane, increasing cell mobility and decreasing cell–cell adhesion capabilities (21). EMT is vital in MIBC progression, as indicated by the upregulation of mesenchymal cell markers (N-cadherin and P-cadherin) and the downregulation of epithelial cell markers (E-cadherin) in MIBC tumors (22). The relationship between the local immune response and the microbiome is exemplified by chronic bacterial infections in the prostate, which are linked to reduced expression of the tumor suppressor protein NKX3.1. NKX3.1 regulates prostatic cell growth and DNA repair. Inflammatory cytokines TNF and IL-1β downregulate NKX3.1, increasing susceptibility to oxidative DNA damage. Loss of NKX3.1 (in mice) can lead to prostatic intraepithelial neoplasia and, in combination with Pten loss, prostate cancer. The cause of prostatic inflammation isn’t attributed to a specific organism but likely involves various species over time (23–26). Inizio modulo To date, the specific mechanism is not known, but there is no singular pathway for carcinogenesis. Instead, each mechanism contributes mutations and abnormalities to the cells, thereby promoting cancer progression.

Predictive tests based on UMB compositions have been proposed, especially involving 16S rRNA sequencing (11, 13), although they have limitations like the inability to detect bacteria at the species level or nonbacterial microorganisms such as viruses and fungi (17). Chipollini et al. (27) found enrichment in unique bacterial communities in invasive bladder cancer patients, suggesting potential for a microbiological signature in high-risk disease. Predictive tests could also help identify non-muscle invasive bladder cancer patients who could benefit from BCG immunotherapy (2). However, UMB signature studies require caution due to issues related to sample collection, biological sample management, and factors like age, menopausal status, sex hormones, and body mass index.

## 2 Methods

A literature review identified relevant studies on urinary microbiome and its association with urologic cancers, mainly bladder cancer, prostate cancer and renal cell carcinoma. PubMed was used as the database, and the collected studies formed the basis for a narrative analysis of the literature published in the last 5 years. We used the following keywords: urinary microbiome, prostate cancer, bladder cancer, renal cell carcinoma, penile cancer, testicular cancer.

## 3 Results

### 3.1 Prostate cancer

Even though it is not fully demonstrated and understood, the main mechanisms by which microbiome could promote prostate cancer seem to be chronic systemic inflammation and immune modulation (28). Cancerous prostate tissue contains bacterial DNA, unlike healthy tissue. Microbial infection weakens the prostate’s natural defenses, causing epithelial disruption, loss of barrier function and persistent inflammation. Although no specific organism is identified as the main cause of prostate inflammation, the urinary tract is a potential source of microorganisms that may enter the prostate. In the last years some studies have tried to investigate which pathogens could be involved in the pathogenesis of prostate cancer. The studies published so far, differentiate for the type of tissue/fluid analyzed (**Table 1**). In one study conducted by Cavarretta et al. (33), the microbiome profile of 16 radical prostatectomy specimens was analyzed. Additionally, two separate studies by Shrestha (34) and Alanee (35) concentrated on differences in urine samples from patients with BPH and PCa. Another study conducted by Yu (36) examined the microbiome in urine samples and in samples of expressed prostatic fluid and seminal fluid obtained through prostatic massage, comparing men with BPH and prostate cancer.

**Table 1:** Summary of analyzed evidence about urinary microbiome and its association with prostate cancer.

Study	Tissue analyzed	Group of patients	Association proposed	Bacteria increased in cancer group	Sample size
Alanee et al, 2019 (35)	Urine sample	Benign prostate biopsies vs prostate cancer	Difference in Increased bacteria species	*Veillonella, Streptococcus, and Bacteroides*	PC patients (n=14) Healthy (n=16) (30 urine sample, 30 fecal sample)
Cavarretta et al, 2017 (33)	Prostate specimen	Radical prostatectomy patients	Increased bacteria species	*Staphylococcus Spp*	PC patients (n=16)
Shrestha et al, 2018 (34)	Urine sample	Benign prostate biopsies vs prostate cancer	Difference in Increased bacteria species	*Streptococcus, Anaerococci, Actinobaculum, Varibaculum*, *Propionimicrobium lymphophilum*	PC patients (n=65)
Yu et al, 2015 (36)	Urine sample and expressed prostatic secretions	BPH and prostate cancer	Differences in Increased bacteria species	*Bacteroidetes*, *Alphaproteobacteria, Firmicutes, Lachnospiraceae, Propionicimonas, Sphingomonas, and Ochrobactrum*	PC patients (n=13) BPH (n=21)

In Cavarretta’s study (33), Staphylococcus Spp. were found in higher representation in pathological specimens. Conversely, in Shretsha’s work (34), the clustered group of bacteria species that proportionally had more cancer samples included Streptococcus anginosus, Anaerococcus lactolyticus, Anaerococcus obesiensis, Actinobaculum schaalii, Varibaculum cambriense, and Propionimicrobium lymphophilum. In Alanee’s study (35), the species Veillonella, Streptococcus, and Bacteroides were found to be more abundant. On the other hand, in Yu’s research, an increased presence of Bacteroidetes bacteria, Alphaproteobacteria, Firmicutes bacteria, Lachnospiraceae, Propionicimonas, Sphingomonas, and Ochrobactrum was observed in the PCa group compared to the BPH group (36).

When it comes to the most frequently identified pathogens, comparing these studies can be challenging, even though all of them have discovered significant differences between the group of individuals with prostate cancer (PCa) and the control group. This complexity arises because, as previously explained, the fluids and tissues analyzed in these studies vary. Despite the inherent limitations associated with different sample types (prostate specimens, urine, prostatic secretions), some bacteria, such as Streptococcus spp and Propionimicrobium, appear to recur in two of these research papers. Conducting additional studies that involve comparing samples from urine, prostate tissue, and secretions obtained from the same patient could provide further clarity regarding which uropathogens might be implicated in the pathogenesis of prostate cancer.

**Table 2**; **Figure 1** summarize evidence about urinary microbiome and its association with prostate cancer.

**Table 2:** Summary of analyzed evidence about urinary microbiome and its association with bladder cancer.

Study	Association proposed	Most involved bacteria	Lower abundance	Other	Sample size
Chipollini (27)	Predominance by single organisms	Bacteroidesa, Faecalbacterium			Healthy (n=10) urothelial carcinoma (n=38)
Zeng (11)	more diversity	Micrococcus, Brachybacterium			Healthy (n=19) BC (n=62)
Wu (13)	increased bacterial richness	Acinetobacter, Escherichia, Shigella, Staphylococcus, Streptococcus, Aeromonas, Bacteroides, Lactobacillus, Serratia, Proteus, Laceyella, Fusobacterium	Serratia, Proteus, and Roseomonas		Healthy (n=18) BC (n=31)
Mai (17)	More abundance	Acinetobacter, Klebsiella			BC (n=24)
Liu (29)	More abundance	Acinetobacter, Cupriavidus spp., Brucellaceae, Anoxybacillus, Escherichia-Shigella, Geobacillus, Pelomonas, Ralstonia, Sphingomonas	Lactobacillus, Prevotella, Ruminococcaceae		Normal tissue (n=12) BC tissue (n=22)
Bi (30)	More abundance	Actinomyces	Lactobacillus		Healthy (n=26) BC (n=29)
Hussein (2)	More abundance	Actinomyces, Achromobacter, Brevibacterium, Brucella		MIBC: Hemophilus and Veillonella Positive response to BCG treatment: Serratia, Brochothrix, Negativicoccus, Escherichia-Shigella, Pseudomonas	Healthy (n=10) BC (n=43)
Popovic (31)			Veillonella, Streptococcus, Corynebacterium		Healthy (n=11) BC (n=12)
Pederzoli (32)	More abundance	Klebsiella			Healthy (n=59) BC (n=49)

**Figure 1:**
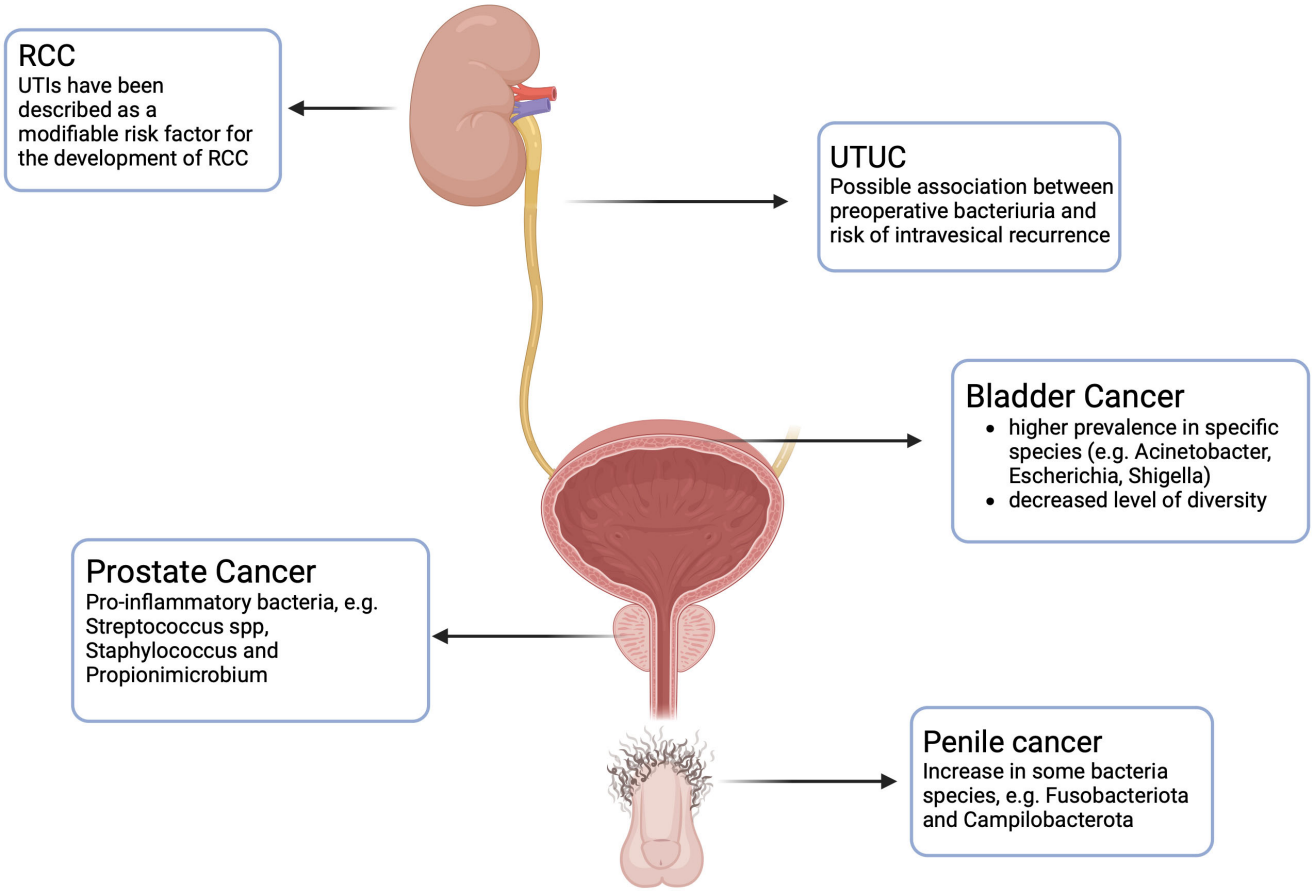
Urinary microbiome and its association with urologic cancers. RCC: Renal Cell Carcinoma. UTUC: Upper Urinary Tract Urothelial Cell Carcinoma. Created with BioRender.com.

### 3.2 Bladder cancer

The studies examining bladder cancer and its relationship with the UMB primarily suggest two main types of associations: either a higher prevalence in specific species or an elevated level of diversity.

Chipollini et al. (27) provide evidence of a reduction in both species’ richness and evenness in the urine of bladder cancer Patients, suggesting a higher probability of a dominant presence of an individual organism.

Acinetobacter is found to be more prevalent in patients with bladder cancer, as described in a study by Mai et al. (17). In a similar manner, Liu et al. (29) revealed higher relative abundances of Acinetobacter in cancerous compared to normal tissues, also with Cupriavidus spp., Brucellaceae, Anoxybacillus, Escherichia-Shigella, Geobacillus, Pelomonas, Ralstonia, and Sphingomonas. Lower relative abundances of the microbial genera Lactobacillus, Prevotella, as well as Ruminococcaceae was observed. Hussein et al. (2) found significant differences in beta-diversity, with Actinomyces, Achromobacter, Brevibacterium, and Brucella being significantly more abundant in urine samples from bladder cancer patients. These findings are partially consistent with those reported by Bi et al. (30), in which Actinomyces had a higher abundance in bladder cancer patients, being other four genera of bacteria more prevalent in healthy controls (Streptococcus, Bifidobacterium, Lactobacillus, Veillonella). Particularly notable was the higher prevalence of Lactobacillus in healthy individuals, a bacterium that has been shown to be a component of the microbiome and to confer protective effects against tumors in various organ systems, including gastrointestinal tumors (37) and gynecological tumors (38). Pederzoli et al. (32) found Klebsiella enrichment in urine of females with bladder cancer, similarly to a previous UMB study (17). Notably, Klebsiella’s colibactin toxin may cause direct DNA-strand damage, leading to genomic instability (39).

Zeng et al. (11) describe an increased bacterial richness index (Observed Species index, Chao1 index, Ace index; all P < 0.01) in cancer group compared to non-neoplastic group. Furthermore, in patients with NMIBC following TURBT, it was observed that the recurrence group displayed significantly greater alpha diversity when compared to the non-recurrence group. Additionally, higher alpha diversity was associated with a shorter time to recurrence.

Similarly, Wu et al. (13) noted elevated bacterial richness levels (including the Observed Species, Chao1, and Ace indexes) along with concurrent enrichment in certain bacterial genera (such as Acinetobacter, Anaerococcus, and Sphingobacterium), and a reduction in others (like Serratia, Proteus, and Roseomonas) when comparing the cancer group to the non-cancer group.

Pederzoli et al. showed that the UMB shares over 80% of the bacterial families found in the paired bladder tissue, indicating that the UMB can serve as a reliable representation of the tissue bacterial environment (32).

On the other hand, Mansour et al. (40) demonstrated a higher presence of certain species (Akkermansia, Bacteroides, Clostridium, Enterobacter and Klebsiella) in bladder tissues compared to the urine.

When comparing NMIBC and MIBC patients, Hussein et al. (2) discovered higher Hemophilus and Veillonella levels in MIBC patients’ urine, while Cupriavidus predominated in NMIBC patients. This aligns with Oresta et al. (41) study, where high-grade NMIBC and T2 tumor patients had more Veillonella in their urine samples and reduced Bifidobacterium and Ruminococcus 1, both of which have anti-inflammatory roles in mucosal homeostasis (42, 43). In contrast, Popovic et al.’s study (31) found Veillonella, along with Streptococcus and Corynebacterium, as the most common bacteria in healthy individuals.

The concept that certain bacteria may offer protection against cancer is notably apparent in urinary bladder cancer. This is unique as it is the only cancer treated with live microorganisms, specifically Mycobacterium bovis - Bacillus Calmette-Guérin (BCG) (44). BCG is believed to function by binding to fibronectin and integrin α5β1, subsequently triggering an immune response (45, 46). It is conceivable that, similarly to BCG, specific commensal bacteria naturally inhabiting a healthy bladder may serve in tumor surveillance or confer different beneficial effects (31). Additionally, the microbiome was proposed to influence responses to adjuvant BCG therapy and systemic immunochemotherapy in individuals with high-risk or advanced bladder cancer cases (2, 47).

**Table 1**; **Figure 1** summarize evidence about urinary microbiome and its association with bladder cancer.

### 3.3 Upper urinary tract urothelial cell carcinoma

The evidence regarding the association between UTUC and urinary microbiome is still very limited, and there are few studies on the topic. Fukushima et al. (48) in their study investigated the effect of perioperative bacteriuria and pyuria on intravesical recurrences in patients with UTUC undergoing radical nephroureterectomy and they found that bacteriuria and pyuria independently predicted a decreased risk of intravesical recurrences (**Figure 1**). Since serial cystoscopies for follow-up are costly and create discomfort for the patient, being able to stratify patients based on preoperative parameters can be useful in understanding which patients are at low risk of recurrence, thus avoiding such stringent follow-up. A different result was obtained by Liang et al., who instead demonstrated that preoperative pyuria among UTUC patients undergoing radical nephroureterectomy was significantly associated with advanced pathological tumor stage and worse survival (49).

The association between local and systemic inflammation and cancer is still controversial. While certain inflammatory and immune responses exhibit anti-tumor activity, inflammation itself can also promote cancer progression. A heightened preoperative CRP level is indicative of a reduced survival and worst prognosis for patients with UTUC (50). Unlike the studies of bladder urothelial cancer, there is no literature regarding the abundance or specific differences of urinary microbiome in patients with upper tract urothelial cancer compared to controls.

### 3.4 RCC

The role of UB in the development of renal cell carcinoma (RCC) is still a debate. An association between prior UTIs and RCC is still unclear, even if UTIs have been described as a modifiable risk factor for the development of RCC (51) (**Figure 1**). Further studies are necessary to clarify the presence of UB in the kidney tissue and its role in the development of RCC.

### 3.5 Microbiota and penile cancer

A recent study conducted by De Deus et al. (52) endeavored to delineate the presence of a microbiome in penile carcinoma tissue. As in other studies previously mentioned, the 16S rRNA was analyzed in both tumor and non-tumor adjacent tissues to assess the presence of different pathogens. They found that Fusobacteriota and Campilobacterota were the two species significantly increased in tumors compared to non-tumor tissues (**Figure 1**).

Furthermore, how penile microbiome can modulate immune response is already well known in other circumstances, as reported by Onywera et al. (53). In fact, changes in the microbiota after circumcision can lead to altered susceptibility to HIV and HPV infection.

These studies represent a starting point to explore the role of microbiome in penile cancer. However, further works are required to elucidate the potential role of microbiome in the pathogenesis of this condition and its implications for developing prevention strategies and treatment modalities.

## 4 Conclusions

The concept of the urinary microbiome is a recent development with potential applications in urologic tumor diagnosis, risk assessment, and treatment. While our analysis of several studies has yielded conflicting outcomes in some instances and inconclusive findings in others, available evidence indicates that certain bacteria may actively contribute to the initiation and progression of tumors. Moreover, these bacteria may also have a role in influencing the response to therapy through immunomodulation. Additional research is required to comprehensively define the characteristics of a healthy urinary microbiome, identify dysbiosis, and understand its potential impact on tumorigenesis and the host’s response.

## Author contributions

GRa: Writing – original draft, Writing – review & editing. EB: Writing – original draft, Writing – review & editing. TC: Writing – original draft. GRe: Writing – original draft. GB: Writing – review & editing. GN: Writing – review & editing. MI: Writing – review & editing. AM: Writing – review & editing. FM: Writing – review & editing. FZ: Writing – review & editing.
